# Cardiovascular magnetic resonance and transesophageal echocardiography in patients with prosthetic valve paravalvular leaks: towards an accurate quantification and stratification

**DOI:** 10.1186/s12968-021-00722-7

**Published:** 2021-03-22

**Authors:** Maciej Haberka, Magdalena Malczewska, Piotr Pysz, Michał Kozłowski, Wojciech Wojakowski, Grzegorz Smolka

**Affiliations:** 1 Department of Cardiology, Medical University of Silesia, Ziołowa 45/47, 40-635 Katowice, Poland; 2 Department of Cardiology and Structural Heart Diseases, Medical University of Silesia, Katowice, Poland; 3 Department of Cardiac Rehabilitation, Treatment and Rehabilitation Center, Long-Term Care Hospital, Jaworze, Poland

**Keywords:** Cardiovascular magnetic resonance, Echocardiography, Paravalvular leak, Valve prosthesis

## Abstract

**Background:**

Objective assessment of prosthetic paravalvular leak (PVL) is complex and challenging even in transesophageal echocardiography (TEE). Our aim was to assess the value of cardiovascular magnetic resonance (CMR) in quantifying PVL in aortic (AVR) or mitral valve (MVR) replacement.

**Methods:**

Thirty-one patients (62 ± 15.1 years, 63% males) with a preliminary diagnosis of significant PVL (AVR, n-23; MVR, n = 8) were recruited. The TEE PVL grading was based on the semi-quantitative (SQ) TEE according to the VARC II PVL classification (%PVL: mild < 10%; moderate 10%–30%; severe > 30%). Non-contrast CMR studies were acquired at 1.5 T with a quantitative approach (phase-contrast velocity encoded imaging). The CMR PVL severity was classified according to regurgitant fraction (RF: (1) mild ≤ 20%, (2) moderate 21%–39%, or (3) severe ≥ 40%).

**Results:**

All patients revealed symptoms of heart failure (71%: New York Heart Association [NYHA] II; 91%: N-terminal pro-B-type natriuretic peptide [NT-proBNP] > 150 pg/ml) and typical cardiovascular disease risk factors. The SQ-TEE results revealed several categories: (1) mild (n = 5; 16%), (2) moderate (n = 21; 67%), and (3) severe (n = 5; 16%) PVL. However, CMR PVL RF reclassified the severity of PVL: (1) mild to moderate (in 80%), (2) moderate to severe (in 47%), and (3) severe to moderate (in 40%). The receiver operating characteristic analysis showed that SQ-TEE and CMR PVL-vol and -RF predicted the upper tertile of NT-proBNP (> 2000 pg/ml) with the best sensitivity for CMR parameters.

**Conclusion:**

The SQ-TEE showed moderate agreement with CMR and underestimated a considerable number of AVR or MVR-PVL.

## Introduction

Prosthetic valve paravalvular leak (PVL) is a rare finding in patients with valve surgical prostheses. PVL is usually an early complication of cardiac surgery or a consequence of infective endocarditis. PVL is detected in up to 10% of patients after aortic valve replacement (AVR) and 17% of patients after mitral valve replacement (MVR) [1, 2]. Although a mild leak may have no clinical consequences, a considerable number of patients with PVL will have persistent symptoms of heart failure (HF), hemolytic anemia, or even a worse clinical prognosis [3]. The surgical treatment for severe PVL is repeat surgery, which is associated with significant mortality and complication rates [4]. Therefore, percutaneous transcatheter closure techniques have become the primary treatment choice in select centers with a highly experienced heart team.

Transthoracic echocardiography (TTE) is the primary imaging tool used in the follow-up of patients with valve prostheses. However, TTE has a high interobserver variability and it is considered a screening method in patients with PVL [5]. Despite this limitation, TTE seems to be a reliable imaging tool in asymptomatic patients with a non-significant PVL. Most patients with HF symptoms or anemia and suspicion of a significant PVL require transesophageal echocardiography (TEE) for better leak identification and grading. However, due to the limitations of the ultrasound technique, neighboring prosthesis, and complex anatomy of the leak channel, it is often difficult to provide precise quantification of PVL on echocardiography [6]. Despite these limitations, TTE and TEE remain imaging modalities of choice in PVL assessment [7].

Cardiovascular magnetic resonance (CMR) is a modern imaging tool with high accuracy and reproducibility in the evaluation of cardiac chamber volume, function, and mass [8, 9]. CMR is considered the gold-standard for these purposes. Although CMR has been validated in the quantitative assessment of some native valve defects [10, 11], the evidence supporting the role of CMR in patients with prosthetic valve PVL is scarce and limited to patients with transcatheter aortic valve implantation (TAVI) [12, 13].

Therefore, our aim was to compare TEE and CMR in PVL quantification related to either AVR or MVR.

## Methods

### Study patients

All the patients with either AVR or MVR and TTE diagnosis of PVL were referred to our center for further diagnostics and treatment. All of the patients had undergone a comprehensive TEE to confirm a PVL versus a transvalvular regurgitation, which was the main inclusion criterion. The exclusion criteria included: (1) any form of prosthesis degeneration resulting in transvalvular regurgitation on TEE; one patient was excluded for transvalvular regurgitation instead of PVL, and one patient was excluded for coexistence of trans- and paravalvular regurgitations, (2) TAVI, (3) contraindications to CMR, and/or (4) high probability of incomplete or nondiagnostic CMR images (decompensated or acute HF symptoms, poorly controlled tachyarrhythmias as seen in one patient), infectious diseases in the previous month.

Finally, 31 patients (62 ± 15.1 years, 63% males) with either AVR-PVL (n = 23) or MVR-PVL (n = 8) were enrolled (2018–2019). A detailed medical history and additional laboratory tests were collected in all the patients, including a complete blood count (CBC), serum lactate dehydrogenase (LDH) and N-terminal pro-B-type natriuretic peptide (NT-proBNP). Hemolysis was identified by serum LDH > 460 U/l, blood hemoglobin < 13.8 g/dl (males) or < 12.4 g/dl (females), and reticulocyte count > 2% with no cause of hemolysis other than PVL [14].

All of the subjects were scheduled for a TEE and non-contrast CMR performed within 1 week at our center. None of the patients were pre-medicated or sedated before or during the TEE and CMR, which would have affected the severity of PVL. All patients had routine clinical follow-up 6 months after their enrollment.

This study was designed as a prospective single-center study and conducted in accordance with the principles of the Declaration of Helsinki and the local ethics committee. All patients provided signed informed consent. This work was supported by the STRATEGMED II grant (National Centre for Research and Development, STRATEGMED2/269488/7/NCBR/2015).

### Echocardiography

All of the TEE studies were performed with a commercially available imaging system (Phillips EPIQ, Philips X7-2t TEE probe, Philips Healthcare, Best, The Netherlands), and each of the examinations followed the European Association of Cardiovascular Imaging (EACVI)/American Society of Echocardiography (ASE) recommendations [15, 16]. The TEE examination was focused on PVL quantification, which was based on the most popular semi-quantitative (SQ) grading system in clinical practice according to the Valve Academic Research Consortium II PVL classification in which the sum of the PVL jets circumferences is divided by the valve circumference (%PVL): (1) mild < 10%, (2) moderate 10%–30%, (3) severe > 30% [17, 18]. All of the TEE images were obtained, stored anonymously, and then analyzed offline by a single observer who was blinded to patients’ clinical characteristics and CMR results.

### Cardiovascular magnetic resonance imaging

The CMR images were acquired on the 1.5 T system (Optima MR450w, General Electirc Healthcare, Waukesha, Wisconsin, USA) with a dedicated phased-array cardiac coil and analysed using a cardiac software (CardiacVX, General Electric Healthcare). The CMR study protocol included a non-contrast examination with a multi-planar cine balanced steady-state free precession (bSSFP) acquisitions and flow visualization using phase contrast (PC) flow imaging. Cardiac chambers volumes and functions were analysed by bSSFP in several planes, including 2- and 4-chambers, orthogonal left ventricular (LV) outflow track, and parallel short-axis planes covering both the atria and ventricles. The typical scan parameters used were time to echo/time of repetition (TE/TR) of 1.9/4.3 ms, slice thickness 4–8 mm (no inter-slice gap), and temporal resolution 30–40 ms. The cine bSSFP planes for the AV prosthesis and ascending aorta were placed perpendicular to the aortic root. Through-plane PC flow imaging was obtained at the slices perpendicular to the axis of flow with the positions just above the AVR and velocity encoding maximum values starting at 200 cm/s. PC imaging was repeated with the modified maximum velocity value and/or the position of the slice to avoid aliasing or artifacts [19]. The AVR-PVL was quantified directly from the PC flow curves obtained at the through-plane most proximal to the prosthesis but without any aliasing. In the case of MVR-PVL, the through-plane PC flow imaging was obtained at the slices in the ascending aorta at the level of the sinotubular junction. The MVR-PVL was quantified indirectly as the difference between LV stroke volume (SV), calculated manually from the bSSFP sequences, and forward SV flow in the ascending aorta [10]. The LV and right ventricle (RV) stroke volumes were calculated manually in each patient to provide internal validation of the above described methods.

The severity of either MVR or AVR-PVL was based on regurgitant volume (PVL-vol) and the regurgitant fraction (PVL-RF). The final grading of CMR PVL was classified according to the RF: (1) mild ≤ 20%, (2) moderate 21%–39%, or (3) severe ≥ 40%. No uniform cut-offs and guidelines for the quantification of PVL in CMR can be found, and we used the same criteria as in the most of the previous papers evaluating the utility of CMR for assessing PVL [20].

### Statistical analysis

The results presented in the manuscript or tables are expressed as means (standard deviation) for normally distributed variables, medians (quartiles Q1–Q3) for abnormal distribution or number (percentage). The distribution was tested for the normality with the Kolmogorov-Smironov test. Baseline clinical parameters and the measures were compared between the subgroups using the *t*-tests for the normally distributed continuous variables (Student’s *t*-test); in case of abnormal distribution, the Mann–Whitney U test was used. Associations between parameters were assessed using Pearson or Spearmen correlation analysis depending on the parametric or nonparametric variables. The cut-off values of the parameters of PVL and LV for the prediction of significant LV overload and haemolytic anaemia were determined in Receiver Operating Characteristic (ROC) curve analysis. A value p < 0.05 was considered statistically significant. Statistical analysis was undertaken using Medcalc software (version 19.1, Osten, Belgium).

## Results

### Study group

A total of 31 consecutive patients with either AVR-PVL (n = 23) or MVR-PVL (n = 8) were enrolled into the study (62 ± 15.1 years, 63% males), and all completed a TEE and a non-contrast CMR. Nineteen patients with a mechanical prosthesis and twelve subjects with a bioprosthesis were included. One patient with two bioprostheses (MVR and AVR) and PVL detected in AV prosthesis was included. No deaths occurred during the 6-month clinical follow-up.

The clinical characteristics, symptoms, and baseline parameters are presented in Table 1. All of the study patients revealed symptoms of HF (New York Heart Association [NYHA II] 71%; NT-proBNP 1180 (324–3310) pg/ml) and typical cardiovascular disease risk factors (hypertension [54%], dyslipidemia [80%], diabetes [51%], and chronic kidney disease [53%]). The median time since the cardiac surgery was 3.6 years (2–34 years). Eight (25%) patients had laboratory evidence of hemolytic anemia due to PVL (5 mechanical and 3 biological prostheses).

**Table 1:** Clinical characteristics of the study group

	Mean (SD) or median (Q1–Q3) or No. (%)
Age (years)	62 ± 15
Female/Male	10 (32%) / 21 (68%)
Diabetes	6 (19%)
Dyslipidemia	25 (80%)
Hypertension	20 (64%)
Smoker or ex-smoker	10 (32%)
Prior myocardial infarction	4 (13%)
Body mass index (kg/m^2^)	27.1 ± 4.1
Obesity	7 (22%)
Chronic kidney disease	14 (45%)
Atrial fibrillation	11 (35%)
NYHA class	
II	22 (71%)
III	9 (29%)
CCS class	
I	28 (90%)
II	3 (9%)
Lab tests	
NT-proBNP (pg/ml)	1180 (324–3310)
NT-proBNP > 150 pg/ml	28 (90%)
LDH (U/l)	319 (246–483)
Haemolysis^1^	8 (25%)
Cardiac surgery	
Coronary artery bypass grafting	6 (19%)
Time since the surgery (years)	3.6 (2–34)
AVR—PVL	23 (75%)
MVR—PVL	8 (25%)
Paravalvular leaks and left ventricle remodeling	
Echocardiography	
Multiple leaks	7 (23%)
VC (mm)—major leak	4.9 ± 1.9
Leaks circumference (%)	17 ± 9
Cardiovascular magnetic resonance	
PVL volume (ml)	44 ± 28
PVL regurgitant fraction (%)	36 ± 13
End diastolic volume (ml)	216 ± 112
Ejection fraction < 50%	5 (16%)
Ejection fraction (%)	56.5 ± 14
Mass (g)	177 ± 63

*AVR* aortic valve replacement, *CCS* Canadian Cardiovascular Society Angina Score, *LDH* Lactate Dehydrogenase, *MVR* mitral valve replacement, *NT-proBNP* N-terminal pro-B-type natriuretic peptide, *PVL* paravalvular leak, *VC* vena contracta

^1^Haemolysis defined as serum Lactate Dehydrogenase > 460 U/L and the following: haemoglobin < 13.8 g/dL for males or < 12.4 g/dL for females and reticulocyte count > 2%

### Paravalvular leak in TEE and CMR

TEE studies showed single PVL in 77% of patients. The PVL circumference ranged from 3 to 45% (mean 17 ± 9%) of the sewing ring circumference. The SQ-TEE could be classified into three groups: (1) mild (n = 5; 16%), (2) moderate (n = 21; 67%) and (3) severe (n = 5; 16%) PVL.

**Fig. 1:**
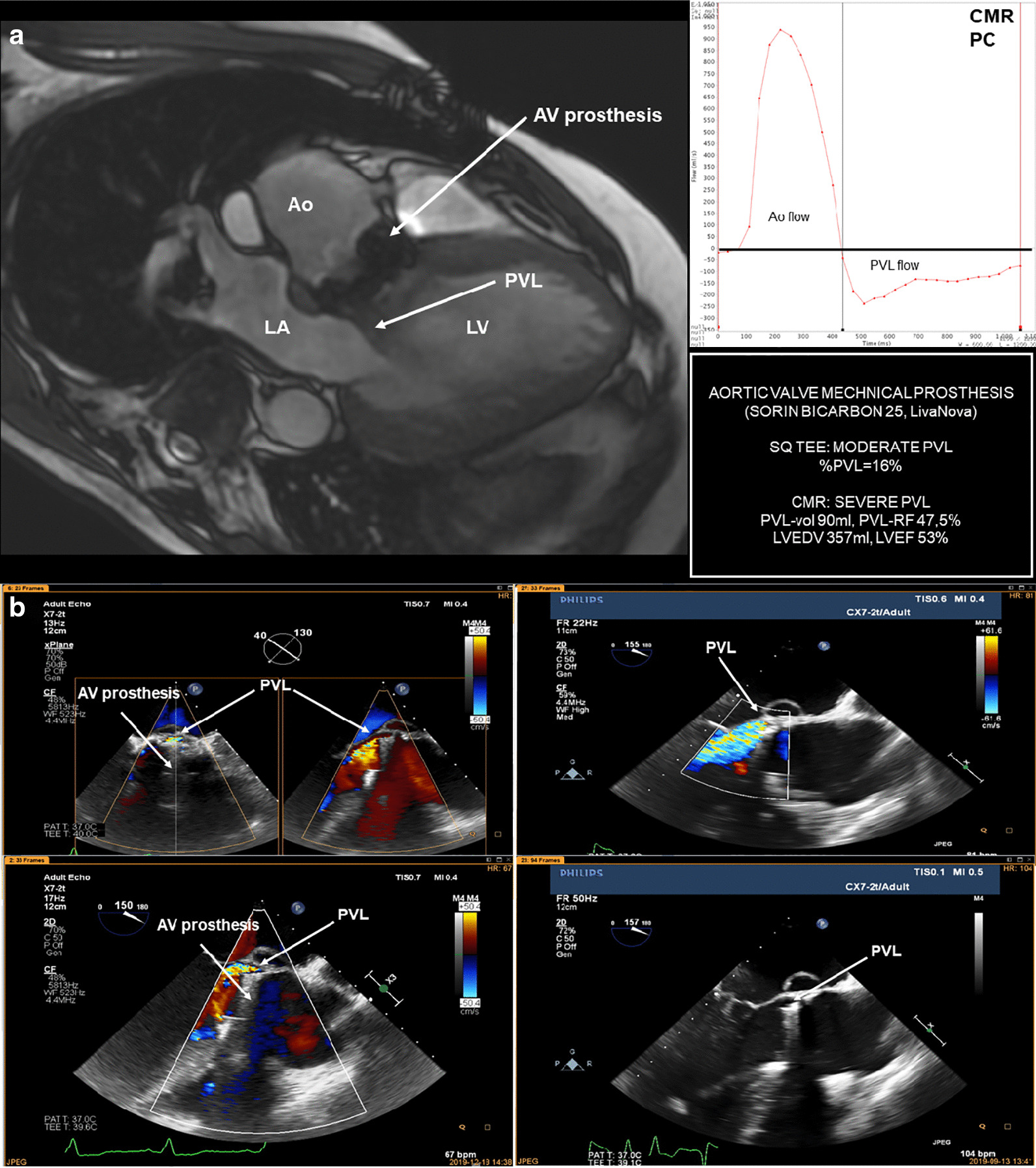
The case of the study patient with a paravalvular leak related to the aortic valve (AV) mechanical prothesis in cardiovascular magnetic resonance (**a**) and transesophageal echocardiography (**b**). *Ao* aorta; *AV* aortic valve; *EDV* end-diastolic volume; *EF* ejection fraction; *LA* left atrium; *LV* left ventricle; *PVL* paravalvular leak; *%PVL* the percentage of the PVL jet circumference related to the prosthetic valve circumference; *PVL-vol* paravalvular leak volume; *PVL-RF* paravalvular leak regurgitation fraction; *SQ* semi-quantitative

**Fig. 2:**
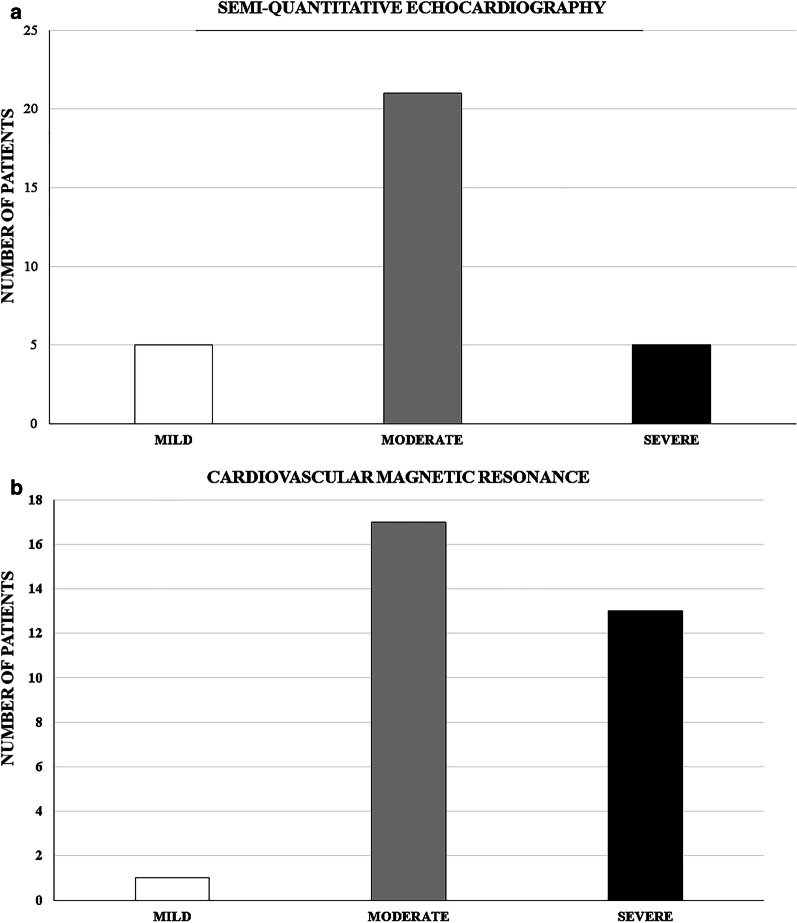
Number of study patients and the severity of paravalvular leak in semi-quantitative echocardiography (**1a**) and cardiovascular magnetic resonance (**1b**)

The CMR PVL-vol ranged from 15 to 120 ml (44 ± 28 ml), and PVL-RF ranged from 16 to 65% (mean 36% ± 13%). The SQ-TEE %PVL revealed a moderate associations with CMR PVL vol (r = 0.6; p < 0.01) and RF (r = 0.55; p < 0.01). Finally, PVL-RF quantified in the CMR reclassified the severity of PVL compared to SQ-TEE: (1) mild to moderate (in 80%), (2) moderate to severe (in 47%), and (3) severe to moderate (in 40%) as shown in Table 2 and Fig. 1. Overall, half of the cases (n = 14; 53%) with a mild or moderate PVL on SQ-TEE were reclassified one grade or higher after CMR (Fig. 2a, b).

**Table 2:** Paravalvular leak grading in transesophageal echocardiography and cardiovascular magnetic resonance

	Transesophageal echocardiography			
Cardiovascular magnetic resonance		Mild	Moderate	Severe
	Mild	**1**	X	X
	Moderate	4	**11**	2
	Severe	X	10	**3**

Bold values indicate consistent grading in both transesophageal echocardiography and cardiovascular magnetic resonance

Patients were divided into two subgroups based on the SQ-TEE %PVL. Patients with %PVL above the median showed significantly higher NT-proBNP compared to those below the median (6349 pg/ml [1688–10,709)] versus 482 pg/ml [303–1933]; p < 0.01).

Patients with PVL-RF in CMR above the median also revealed significantly increased NT-proBNP compared to subjects with PVL-RF below the median (3310 pg/ml [1338–6349] versus 329 pg/ml [176–1905]; p < 0.01).

The NT-proBNP serum levels showed significant associations with CMR PVL-RF (r = 0.65; p < 0.001), CMR PVL-vol (r = 0.5; p < 0.01), and TEE SQ-PVL (r = 0.45; p = 0.02). However, neither CMR PVL-RF nor PVL-vol revealed any associations with the vena contracta of the PVL on TEE (p = ns).

The study group was divided into subgroups of patients with PVL around mechanical or biological prostheses. No differences in the associations between SQ-TEE %PVL and CMR PVL-vol or CMR PVL-RF in either mechanical or biological prostheses were found (Table 3).

**Table 3:** Paravalvular leaks in biological and mechanical prostheses

	Biological prosthesis No. (%)	Mechanical prosthesis No. (%)	p
Paravalvular leak—prosthesis	12 (38%)	19 (62%)	0.2
Aortic valve prosthesis	10 (32%)	13 (42%)	0.6
Mitral valve prosthesis	2 (6,5%)	6 (19,5%)	0.3
Hemolytic anemia	3 (25%)	5 (26%)	0.95
Similar grade of PVL severity in SQ-TEE and CMR	7 (58%)	9 (47%)	0.67
Association between paravalvular leak measures in TEE and CMR			
SQ-TEE %PVL and CMR PVL-vol	r = 0.78; p = 0.01	r = 0.64; p = 0.01	0.5
SQ-TEE %PVL and CMR PVL-RF	r = 0.62; p = 0.04	r = 0.68; p = 0. 01	0.8

*CMR* cardiovascular magnetic resonance, *PVL* paravalvular leak, *PVL-RF* paravalvular leak regurgitation fraction, *PVL-vol* paravalvular leak volume, *SQ-TEE* semi-quantitative transesophageal echocardiography

The association between SQ-TEE and CMR PVL-vol showed no significant differences in patients with MVR-PVL compared to AVR-PVL (r = 0.83 versus r = 0.56; p = 0.26). However, a significant association between SQ-TEE and CMR PVL-RF was found only in AVR (r = 0.62; p < 0.01) and not in MVR-PVL (r = 0.6; p = 0.1). The rates of concordant severity of PVL assessed in SQ-TEE and CMR were similar between MVR and AVRs (50% versus 47%, p = 0.88).

Eight cases of hemolytic anemia (25%) were analyzed, and those patients’ PVLs were classified in SQ-TEE as mild (patient = 1), moderate (n = 6), and severe (n = 1). The CMR quantifications in those patients revealed mostly severe (n = 5) or moderate (n = 3) PVL.

### Paravalvular leak and left ventricle

LV dimensions and systolic function were assessed in all patients in CMR. The median for LV ejection fraction (LVEF) was 59% (52%–65%), and five patients with systolic dysfunction (LVEF < 50%) were included in the study. The LV end-diastolic volume (LVEDV) ranged from 111 to 673 ml (mean 183 ml [145–258]) and LV mass from 60 to 343 g (mean 168 g [135–213]). Both CMR and TEE showed significant associations in LV parameters (LVEDV: r = 0.9; p < 0.001 and LV mass: r = 0.7; p < 0.001). However, significant differences between CMR and echocardiography in LVEDV were noted (mean 183 ml [145–258] versus 145 ml [111–190]; p = 0.02) and LV mass (mean 168 g [135–213] versus 247 g [200–318] p < 0.001).

LVEDV quantified by CMR showed a strong association with CMR PVL-vol (r = 0.7; p < 0.001), a moderate correlation with CMR PVL-RF (r = 0.4; p = 0.01), and also an association with %PVL obtained in TEE (r = 0.5; p = 0.01).

### Prediction of significant PVL

Analysis of receiver operating characterisitic (ROC) curve was performed to find the optimal parameters to predict the greatest cardiac overload and hemolysis.

The ROC analysis showed that %PVL and CMR PVL-vol and PVL-RF predicted the upper tertile of NT-proBNP (> 2000 pg/ml) in our study group with the best sensitivity for CMR parameters (Table 4). However, none of the CMR LV parameters (EDV, LVEF, ass) predicted the upper tertile of NT-proBNP (p = ns).

**Table 4:** The ROC analysis in the prediction of the upper tertile of N-terminal pro-brain natriuretic peptide (NTproBNP > 2000 pg/ml)

PVL quantification	AUC (SE)	p	Optimal value	Sensitivity (%)	Specificity (%)	PPV (%)	NPV (%)
TEE: PVL circumference (%)	0.746 (0.116)	0.03	21	62	87	71	82
CMR: PVL volume (ml)	0.791 (0.09)	0.001	30	100	70	57	100
CMR: PVL regurgitant fraction (%)	0.79 (0.08	0.001	27,5	100	65	53	100

*AUC* area under the curve, *CMR* cardiovascular magnetic resonance, *NPV* negative prediction value, *PVL* paravalvular leak, *PPV* positive prediction value, *ROC* receiver operating characteristics, *SE* standard error, *TEE* transesophageal echocardiography

Moreover, the ROC analysis revealed that all the imaging parameters used in our study, including TEE (%PVL) and CMR (PVL-vol, PVL-RF, LV EDV, LVEF, and mass), failed to provide a statistically significant prediction for PVL-related hemolysis.

## Discussion

Our prospective study presents important findings with respect to the added value of CMR for diagnostic grading and risk stratification in symptomatic patients with AVR or MVR and PVL. First, SQ-TEE showed a moderate association with CMR PVL-vol and RF and underestimated the CMR severity of PVL in half of these cases. Those observations were not dependent on the type (mechanical or biological) or position (AVR or MVR). Both SQ-TEE and CMR PC quantification parameters of PVL were associated with NT-proBNP. However, CMR PVL-vol and PVL-RF, but not LV parameters, showed the strongest correlation and the best prediction for the severely increased NT-proBNP. As expected, the CMR PVL-vol revealed the strongest association with LV enlargement. Finally, none of the TEE or CMR parameters predicted PVL-related hemolysis.

To the best of our knowledge, we present the first study evaluating the added value of CMR to SQ-TEE in patient quantification and stratification with a PVL related to MVR or AVR.

### Echocardiography and CMR imaging in PVL

Pflaumer et al. published a first case report (2005) of a severe AVR PVL confirmed on CMR, which was underestimated on echocardiography [21]. There are only a few studies, which compared the utility of CMR and echocardiography in patients with PVL. However, the study groups were limited only to patients with PVL related to TAVI and used mostly TTE as a comparison modality for CMR. Although the study by Crouch et al. enrolled patients with either surgical aortic valve replacement (SAVR) or TAVI, the patients were all analyzed as one group [22]. Orwat et al. showed that TTE has only a moderate agreement with CMR and strongly underestimated the degree of PVL in the TAVI group [23]. Hartlage et al. also showed that CMR led to a reclassification of the severity of TAVI-related PVL in most cases compared to TTE. Their results suggest that SQ-TTE overestimated the degree of PVL in a considerable number of cases. However, the quality of TTE itself seems not sufficient for PVL grading, especially when it is a retrospective design and used only in symptomatic, non-consecutive patients [20]. The meta-analysis by Papanastasiou et al. reviewed seven studies on the utility of TTE and CMR in patients with post-TAVI PVL [13]. They found a significant disconcordance between TTE and CMR in grading of PVL. In most studies, TTE only had sufficient power to distinguish none or mild and moderate or severe PVL. Moreover, most of the TAVI studies showed that TTE underestimated the severity of TAVI-PVL, which is in line with our results in the group of patients with MVR and AVR [5, 20, 24].

Underestimation of PVL in echocardiography may also underestimate our interpretation of patient symptoms, result in suboptimal pharmacotherapy, and limit the number of patients scheduled for percutaneous transcatheter closure or repeat surgery. This misclassification of AV-PVL severity may explain worse outcomes in patients with even mild PVL in TTE [6, 25]. In our study group, a majority of patients with mild PVL in SQ-TEE were found to have a moderate PVL on CMR. It was demonstrated that patients with at least moderate PVL in TAVI prosthesis confirmed with CMR revealed worse outcomes and clinical prognosis [20, 26].

We do not have a clinical follow-up in our study group yet, so we cannot relate our imaging parameters to patients’ prognosis. Instead, we used NT-proBNP, which is an important marker of clinical prognosis, cardiac overload and wall stress, especially in patients with PVL. While parameters acquired in both modalities were associated with NT-proBNP, CMR-derived quantification parameters showed stronger correlation coefficients with this natriuretic peptide. Schewel et al. found that an increased in NT-proBNP that was greater than 1640 ng/l in patients with post-procedural PVL was associated with significantly increased rate of death in a follow-up [27]. In our study, CMR PVL-vol > 30 ml and PVL-RF > 27.5% showed a very high sensitivity in predicting NT-proBNP levels with a similar cut-off to the study by Schewel et al. and a worse prognosis [27]. The lack of association between LV volume or function in CMR and NT-proBNP was unexpected and suggests that increased NT-proBNP values add to patient’s symptoms and the severity of PVL and not to the LV dysfunction. Given the time since the cardiac surgery and chronic type of PVL in our patients, CMR PVL-vol showed the strongest association with the LV enlargement. However, CMR PVL-RF might be a better estimate of PVL in subjects with an acute PVL or those in their early post-surgical periods.

Eight (25%) patients had laboratory evidence of hemolytic anemia related to PVL. Although CMR led to an upgrade of the class of PVL in most of them, none of the imaging parameters of PVL severity or LV remodeling predicted the PVL-related hemolysis. No other reference reports describe this prediction. This finding suggests that the mechanism behind this phenomenon is complex and that there is no straight association between the severity of leak, shear stress, and the degree of red blood cell damage [28].

The CMR PC technique is based on the assessment of velocities in the selected image plane. Different lengths of valve prostheses used in SAVR or TAVI require using different levels of assessment in the ascending aorta, which might impact the grading of PVL. Therefore, our results are not strictly comparable to the studies assessing PVL related to TAVI [29].

Finally, the great majority of studies used only TTE in comparison to CMR, which has a modest agreement with TEE in the degree of PVL [30]. Although patients with PVL have a multiparametric echocardiography assessment of PVL according to the guidelines [12], a large number of qualitative or SQ parameters still give a wide range of final conclusion in most cases. Therefore, we focused on the SQ-TEE in a further analysis, which is currently the most prevalent classification used in clinical practice. The complex anatomy of leak channels preclude the use of the same echocardiography quantification as in native valve regurgitations. Besides, only a moderate agreement between echocardiography and CMR in those parameters using proximal isovelocity surface area (PISA) [11] was found. Finally, the quality of images (prosthesis) obtained in particular patient affects the measures of severity of PVL, especially in echocardiography. It depends on the type and location of prosthesis and exact anatomy of leak channel. Our results showed no differences in the strength of associations between SQ TEE and CMR in patients with either mechanical prosthesis or bioprosthesis.

### Study limitations

Our study group included mostly patients with AVRs-related PVL with a minority of MVR-PVL. The total number of study patients was too low to provide a separate analysis for both subgroups. The clinical characteristics of the study patients is complex and reflects the real clinical practice. However, although a prosthetic PVL was a main cause of HF symptoms and LV remodeling, it was not possible to separate the minor effects of other factors (such as long-standing native valve defect prior to surgery) or comorbidities. We only had a 6-month clinical follow-up with vital status of study patients (alive or dead), and this period was not enough to relate our results to clinical prognosis. We did not use three-dimensional echocardiography parameters in this study, which could have improved the compatibility in measurements with CMR. Finally, although CMR is a reference tool for volumetric assessment, no true gold standard for measuring the severity of PVL exists. Moreover, the PC method may also be susceptible to artifacts in close proximity to prosthesis. Finally, quantification of MVR function is always an indirect method that is more prone to errors.

### Conclusions and clinical perspectives

SQ-TEE shows moderate agreement with CMR, and the Valve Academic Research Consortium II classification underestimated a considerable number of AVR or MVR-PVL cases. The LV cavity enlargement assessed by CMR reflects the PVL-vol, and a significant increase in NT-proBNP is related to PVL severity and not to LV remodeling. While there is no true gold standard for the severity of PVL, a non-contrast CMR did appear to show superior prediction for the upper tertile of NT-proBNP (CMR: PVL-vol > 30 ml; sensitivity 100% or RF > 27.5%; sensitivity 100%) compared to SQ-TEE (PVL circumference > 21%; sensitivity 62%). Neither TEE nor CMR parameters are helpful in predicting PVL-related hemolysis.

TEE is a necessary imaging modality for screening, grading, and guiding the percutaneous interventions. However, CMR is a complementary tool, which should be implemented in routine practice in patients with at least moderate PVL and/or difficult, incoherent cases. Finally, a need for evidence supporting new cut-off values for CMR quantification of PVL and optimal time for intervention exists.

## Data Availability

Not applicable.

## Abbreviations

ASE: American Society of Echocardiography
AV: Aortic valve
AVR: Aortic valve replacement
bSSFP: Balanced steady state free precession
CBC: Complete blood count
CMR: Cardiovascular magnetic resonance
EACVI: European Association of Cardiovascular Imaging
HF: Heart failure
LDH: Lactate dehydrogenase
LV: Left ventricle/left ventricular
LVEDV: Left ventricular end-diastolic volume
LVEF: Left ventricular ejection fraction
MV: Mitral valve
MVR: Mitral valve replacement
NT-proBNP: N-terminal pro brain natriuretic peptide
NYHA: New York Heart Association
PC: Phase contrast
PISA: Proximal isovelocity surface area
PVL: Paravalvular leak
RF: Regurgitant fraction
ROC: Receiver operator curve
RV: Right ventricle/right ventricular
SAVR: Surgical aortic valve replacement
SQ: Semi quantitative
SV: Stroke volume
TAVI: Transcatheter aortic valve implantation
TEE: Transesophageal echocardiography
TTE: Transthoracic echocardiography
VC: Vena contracta
